# Nerve Root Sedimentation Sign: Can It Predict the Success for Surgical Intervention in Patients With Symptomatic Lumbar Spinal Stenosis?

**DOI:** 10.7759/cureus.9803

**Published:** 2020-08-17

**Authors:** Siddharth A Badve, Swamy Kurra, Fred H Geisler, Umesh Metkar, Richard Tallarico, William Lavelle

**Affiliations:** 1 Orthopedics Spine Surgery, Lewistown Hospital, Lewistown, USA; 2 Orthopedic Surgery, State University of New York Upstate Medical University, Syracuse, USA; 3 Medical Imaging, College of Medicine at the University of Saskatchewan, Saskatoon, CAN; 4 Orthopedics, The Spine Center at Beth Israel Deaconess Medical Center, Boston, USA

**Keywords:** lumbar spinal stenosis, interspinous spacers, surgical management, nerve root sedimentation sign, long-term clinical outcomes

## Abstract

Introduction: The use of interspinous process devices are less invasive surgical methods designed to manage mild to moderate lumbar spinal stenosis symptoms. Symptomatic relief may not be seen in all patients undergoing this procedure. Magnetic resonance imaging (MRI) parameters have been used to predict the success of clinical outcomes in patients with symptomatic lumbar spinal stenosis for decompressive surgeries. The purpose of this study was to determine the feasibility of using nerve root sedimentation sign to predict mid- to long-term clinical outcomes of patients treated with interspinous spacers for lumbar spinal stenosis.

Methods: This was a retrospective study using prospective multicenter Food and Drug Administration Investigational Device Exemption (FDA IDE) trial (Superion™ and X-STOP®) data. Inclusion criteria were patients treated with interspinous spacers, aged 45 or older with lumbar spinal stenosis at one or more contiguous levels from L1 to L5 and symptoms of neurogenic claudication. Preoperative axial T2 weighted MRI images were used to determine nerve root sedimentation sign. Preoperative, six-week, one- and two-year postoperative clinical outcomes were measured using Oswestry Disability Index (ODI) scores. Clinical outcomes were compared between positive and negative nerve root sedimentation sign groups; p ≤0.05 was considered significant.

Results: This study included n=374 patients; 40 excluded; 334 included (113=positive nerve root sedimentation sign (NRSS) (34%) and 221=negative NRSS (66%)). At six weeks, significant postoperative ODI correction was noted in both groups (p<0.001). No significant differences in ODI scores were identified between groups. A subgroup analysis with MRI image quality grade 3 and certainty determination grade 5, six-week postoperative ODI correction was significant in both groups. Six-week, one- and two-year postoperative ODI scores were greater by 6 points in the positive nerve root sedimentation sign group compared to the negative nerve root sedimentation sign group.

Conclusions: Although satisfactory postoperative improvement occurred in both groups, there were statistically significant differences noted in certain sub-categories. The subgroup analysis indicated MRI image quality and nerve root sedimentation sign certainty of determination may be factors that may aid with planning the surgical management of lumbar spinal stenosis.

## Introduction

Lumbar spinal stenosis is described as narrowing of the spinal canal resulting in compression of the spinal cord and cauda equina. The condition may present as radicular symptoms and neurogenic claudication pain in the lower extremities along with chronic low back discomfort [1,2]. Lumbar spinal stenosis is a common indication for surgery when conservative treatment is ineffective [3-5].

Various studies have evaluated the relationship between symptom severity due to lumbar spinal stenosis and radiological parameters with varying outcomes. The dural cross-sectional area is one parameter that has been evaluated [6-10]. Barz et al. introduced the concept of nerve root sedimentation sign on magnetic resonance imaging (MRI) scans in relation to lumbar spinal stenosis [11]. A positive nerve root sedimentation sign is defined as the absence of normal nerve root sedimentation in at least one or more axial T2 weighted MRI sequence images. 

Tomkins-Lane et al. evaluated the sensitivity, specificity, and reliability of the nerve root sedimentation sign for the diagnosis of lumbar spinal stenosis [12]. The nerve root sedimentation sign was helpful to distinguish between lumbar spinal stenosis and non-symptomatic controls but could not differentiate between lumbar spinal stenosis and low back pain or lumbar spinal stenosis and vascular claudication. Additional studies have indicated the nerve root sedimentation sign may be a potential predicting factor for successful surgical treatment in the management of lumbar spinal stenosis [11,13-15].

The use of interspinous process devices is a relatively recent, less invasive surgical method designed to manage mild to moderate lumbar spinal stenosis symptoms. Symptomatic relief may not be seen in all patients undergoing this procedure [5,16,17]. As typical of Scott's parabola, with the introduction of any technology, the initial enthusiasm utilizing interspinous process devices has gradually waned [18]. A major contributing factor to the marginal success of this device is lack of well-defined indications for their use.

Our objective was to determine the feasibility of using nerve root sedimentation signs in predicting mid- to long-term clinical outcomes (one- and two-year postoperatively) for patients undergoing surgical treatment with interspinous process spacer implantation. The presumptive hypothesis is the assumption that patients with a positive nerve root sedimentation sign will have a better symptomatic improvement after surgical intervention with the implantation of an interspinous device.

## Materials and methods

Data from the prospective multicenter Food and Drug Administration Investigational Device Exemption (FDA IDE) trial of Superion™ (Vertiflex, Carlsbad, CA) and X-STOP® (Medtronic, Minneapolis, MN) for the treatment of lumbar spinal stenosis was utilized for this study. This was an institutional review board-approved trial.

The study included patients from the Superion™ and X-STOP® database, with interspinous process devices implanted as a part of their treatment for lumbar spinal stenosis. Inclusion criteria were patients aged 45 or older and suffering from moderate symptoms of neurogenic intermittent claudication secondary to a confirmed diagnosis of lumbar spinal stenosis at one or two contiguous levels from L1 to L5. The study excluded the L5-S1 level in the evaluation process.

Axial T2 weighted preoperative MRI images were used to find the nerve root sedimentation sign. The images were evaluated by two independent reviewers (fellowship-trained spine surgeons) who were not part of the treatment team. To determine the presence or absence of a nerve root sedimentation sign, the dural sac on axial T2 weighted preoperative MRI images was divided into an anterior and posterior half by an imaginary horizontal line. In a non-stenotic spinal canal, the nerve roots other than the exiting roots tend to sediment in the posterior half of the spinal canal below the equator. In a stenotic spinal canal, the roots are clumped together and located in the anterior and posterior half of the spinal canal. Thus a positive nerve root sedimentation sign is defined as the absence of normal nerve root sedimentation in the posterior half of the spinal canal at least one axial T2 weighted MRI sequence image (in addition to other levels above or below). 

Inconsistency in nerve root sedimentation sign determination by the reviewers, due variations in MRI image quality and disparity in spinal stenosis severity, was a concern. This was addressed by a subclassification of patients with a positive nerve root sedimentation sign. Based on the nerve root sedimentation sign certainty of determination, patients were subclassified as Grades 1 to 5, with higher grades indicating a greater certainty. In addition, based on MRI image quality, the groups were subclassified as Grades 1 to 3, with higher grades indicating better image quality. The classification was based on consensus criteria devised by the senior authors. The image assessment was carried out by two fellowship-trained spine surgeons not involved in the patient treatment. 

**Figure 1:**
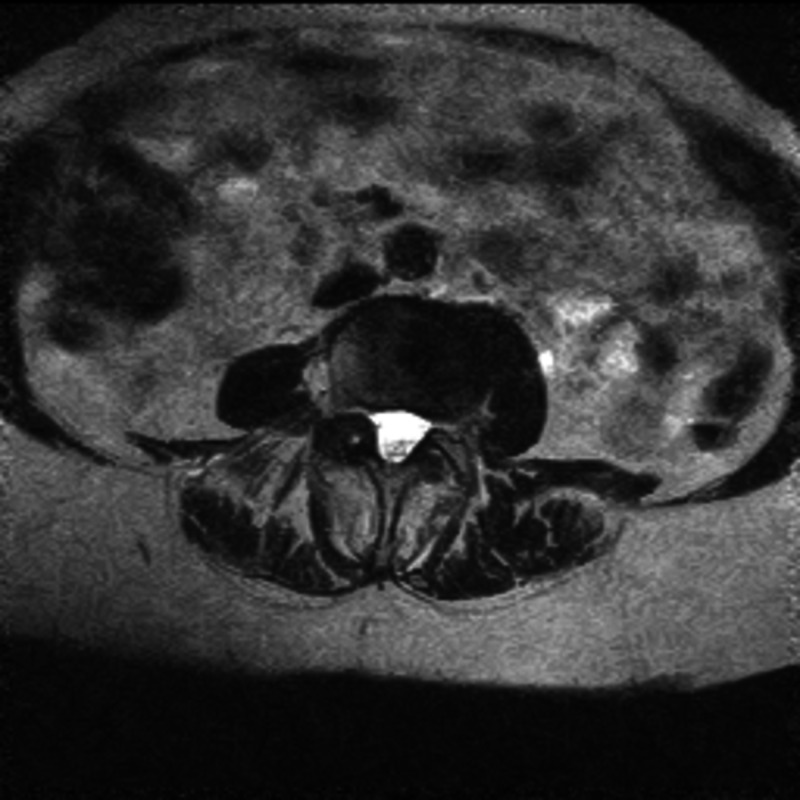
Axial T2 weighted MRI image with negative nerve root sedimentation sign.

**Figure 2:**
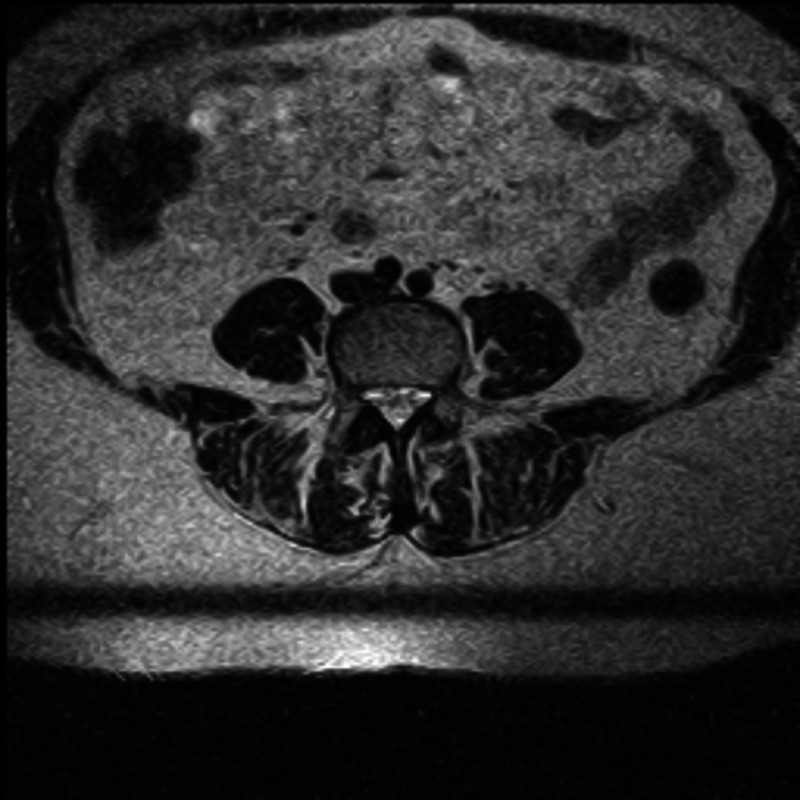
Axial T2 weighted MRI image with positive nerve root sedimentation sign.

Preoperative, six-week, one- and two-year postoperative clinical outcomes were measured using Oswestry Disability Index (ODI) scores. Patients were grouped based on their nerve root sedimentation sign (positive or negative) (Figures 1, 2) and clinical outcomes were compared between the groups.

 

Statistical Analysis:

IBM SPSS Statistics 24 (IBM, Armonk, NY) software was used for the analysis. A paired t-test was used for continuous variables and the chi-square tests were used for categorical variables; p ≤0.05 was considered statistically significant.

## Results

Preoperative MRI imaging sequences from 374 patients were evaluated with only 334 patients included in this analysis. Forty patients were excluded due to incomplete MRI images (N=24) or disagreement between the reviewers about the nerve root sedimentation sign (N=16). The nerve root sedimentation sign agreement rate between the reviewers was 96%.

**Figure 3:**
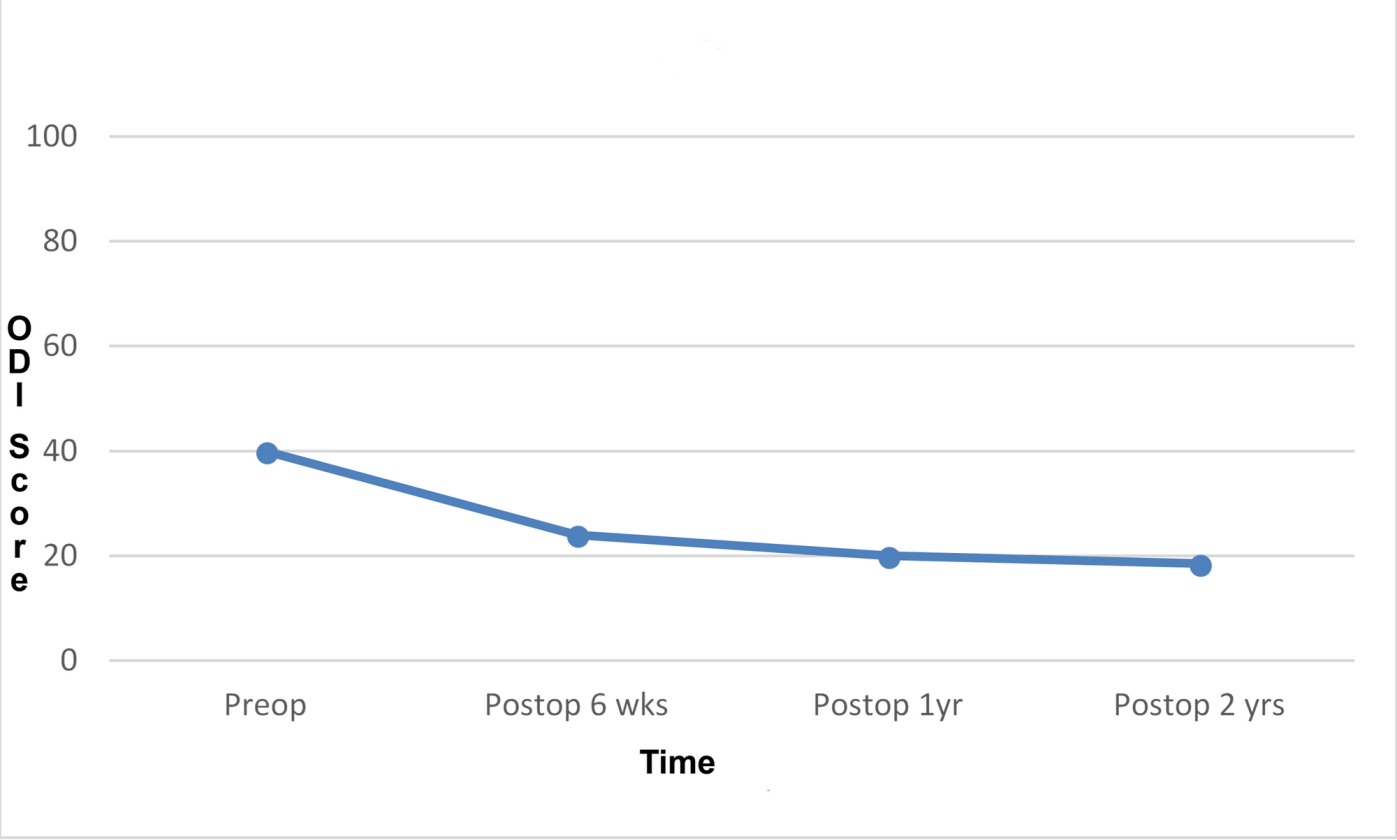
Linear graph representing the changes in Owestry Disability Index (ODI) scores from preoperative to final follow-up.

One hundred fifty-seven (157) patients (47%) received the Superion™ interspinous device and 177 patients (53%) received the X-STOP® device implantation. One hundred thirteen (113) patients (34%) had a positive nerve root sedimentation sign and 221 patients (66%) had a negative nerve root sedimentation sign. The mean preoperative ODI score was 40 (range: 6.7 to 80). At six weeks postoperatively, the mean ODI score was 24 (range: 0 to 80); at one year, mean ODI score was 20 (range: 0 to 66); and at two years, mean ODI score was 18.5 (range: 0 to 77). ODI correction preoperatively to the six-week postoperative value was significant (paired t-test; p<0.001). A significant decrease in mean ODI scores was noted throughout the follow-ups in comparison to the preoperative value (linear regression; p<0.001) (Figure 3).

The mean preoperative patient ODI scores were 39 (range: 9 to 74) with a negative nerve root sedimentation sign and 40 (range: 7 to 72) with a positive nerve root sedimentation sign. At six-week, one-year, and two-year postoperative follow-ups, mean ODI scores for the positive nerve root sedimentation sign group were: 23 (range: 0 to 80), 20 (range: 0 to 66) and 18 (range: 0 to 78); and for the negative nerve root sedimentation sign group the scores were: 25 (range: 0 to 72), 21 (range: 0 to 62) and 19 (range: 0 to 68). No significant differences in ODI scores were identified between the negative and positive nerve root sedimentation sign groups at preoperative, six-week, one-year and two-year postoperative follow-ups (Table 1). ODI corrections from preoperative to six-week postoperative values were significant, in both negative and positive nerve root sedimentation sign groups (paired t-test; p<0.001, p<0.001, respectively).

**Table 1:** Summary of Oswestry Disability Index (ODI) scores comparison between negative and positive nerve root sedimentation sign groups.

	Negative Nerve Root Sedimentation Sign	Positive Nerve Root Sedimentation Sign	P value
n	221	113	
Mean ODI preoperative	39 (range: 9 -74)	40 (range: 7 -72)	0.42
Mean ODI 6-week postoperative	23 (range: 0 – 80)	25 (range: 0 – 72)	0.30
Mean ODI 1-year postoperative	20 (range: 0 – 66)	21 (range: 0 – 62)	0.67
Mean ODI 2-year postoperative	18 (range: 0 – 78)	19 (range: 0-68)	0.61
Device Type	X-STOP® (52%) Superion™ (48%)	X-STOP® (55%) Superion™ (45%)	0.35
Mean Number of Fractures	0.23	0.18	0.46

Subgroups Analyses:

Subgroups were formed based on the reviewers’ nerve root sedimentation sign certainty of determination (Grade 1-5) and MRI image quality (Grade 1-3).

In the patient subgroup with MRI image quality ≥ grade 2 and nerve root sedimentation sign certainty of determination ≥ grade 4, 202 patients had a negative nerve root sedimentation sign and 96 patients had a positive nerve root sedimentation sign. The mean six-week postoperative ODI correction was significant in both groups (paired t-test; p<0.001, p<0.001, respectively). No significant differences were noted in ODI scores between negative and positive nerve root sedimentation sign groups with preoperative, six-week, one-year and two-year postoperative scores, respectively (Table 2).

**Table 2:** MRI image quality Grade ≥2 and certainty Grade ≥4 subgroup Oswestry Disability Index (ODI) scores comparison between negative and positive nerve root sedimentation sign groups.

	Negative Nerve Root Sedimentation Sign	Positive Nerve Root Sedimentation Sign	P value
n	202	96	
Mean ODI preoperative	39 (range: 9-74)	40 (range: 7-72)	0.57
Mean ODI 6-week postoperative	23 (range: 0-80)	25 (range: 0-72)	0.37
Mean ODI 1-year postoperative	20 (range: 0-66)	22 (range: 0-62)	0.46
Mean ODI 2-year postoperative	19 (range: 0-78)	20 (range: 0-68)	0.56
Device Type	X-STOP® (52%) Superion™ (48%)	X-STOP® (53%) Superion™ (47%)	0.5
Mean Number of Fractures	0.24 (range: 0-2)	0.21 (range: 0-2)	0.69

In the patient subgroup analysis with MRI image quality grade 3 and nerve root sedimentation sign certainty of determination grade 5, 116 patients had a negative nerve root sedimentation sign and 61 patients had a positive nerve root sedimentation sign. No significant difference was noticed for preoperative ODI scores between negative nerve root sedimentation sign patients (mean, 38) and positive nerve root sedimentation sign patients (mean, 39), p = 0.45. At six-weeks postoperatively, ODI correction was significant in both negative and positive nerve root sedimentation sign groups (paired t-test; p<0.001, p<0.001, respectively). However, six-week (p=0.012), one-year (p=0.04) and two-year (p=0.039) postoperative ODI scores were statistically higher by six points in the positive nerve root sedimentation sign group compared to the negative nerve root sedimentation sign group (Table 3).

**Table 3:** MRI image quality Grade 3 and certainty Grade 5 subgroup Oswestry Disability Index (ODI) scores comparison between negative and positive nerve root sedimentation sign groups.

	Negative Nerve Root Sedimentation Sign	Positive Nerve Root Sedimentation Sign	P value
n	116	61	
Mean ODI preoperative	38 (range: 9-70)	39 (range: 7-72)	0.45
Mean ODI 6-week postoperative	22 (range: 0-80)	28 (range: 0-72)	0.012
Mean ODI 1-year postoperative	18 (range: 0-66)	24(range: 0-62)	0.04
Mean ODI 2-year postoperative	16 (range: 0-51)	22(range: 0-68)	0.039
Device Type	X-STOP® (58%) Superion™ (42%)	X-STOP® (48%) Superion™ (52%)	0.12
Mean Number of Fractures	0.32	0.28	0.70

## Discussion

Our study attempted to use the nerve root sedimentation sign to predict differences in the clinical outcomes in patients with lumbar spinal stenosis treated with interspinous device implantation. Clinical improvement was noticed in both abnormal and normal nerve root sedimentation signs in patients who were managed by interspinous process devices for symptomatic lumbar stenosis. 

We identified a positive correlation between lack of a nerve root sedimentation sign (positive nerve root sedimentation sign) in preoperative MRI images and long-term clinical outcomes in interspinous process spacer implanted patients in certain subgroups. Although clinical improvement was statistically different between abnormal and normal nerve root sedimentation patients in the select sub-groups, it may not be clinically meaningful.

Based on a patient subgroup analysis with MRI image quality grade 2 and nerve root sedimentation sign certainty of determination grade 4, no significant difference was noted in ODI scores between negative and positive nerve root sedimentation sign groups preoperatively, six-week, one-year, and two-year postoperative scores, respectively. However, the patient subgroup analysis with MRI image quality grade 3 and nerve root sedimentation sign certainty of determination grade 5, six-week, one-year, and two-year postoperative ODI scores were greater by six points in positive nerve root sedimentation sign patients compared to those with negative nerve root sedimentation sign.

Interspinous spacers are a more recent alternative to conservative and conventional surgical treatments for lumbar spinal stenosis. As the experience with the use of these devices has widened, the implant designs have evolved and the understanding for the indications, efficacy and adverse events has greatly improved [5,16,17].

Various studies have evaluated the relationship between symptom severity due to lumbar spinal stenosis and radiological parameters with varying outcomes. The dural cross-sectional area is one parameter that has been evaluated. These studies identified an inconsistent and contradictory relationship between the dural cross-sectional area as an indicator of the severity of spinal stenosis and the nature of patients’ symptoms along with the extent of functional impairment [6-10].

Schizas et al. described a classification of spinal stenosis based on neural tissue impingement [19]. The morphology of the dural sac including the cerebrospinal fluid and nerve root ratio on T2 axial images was graded and this formed the basis for surgical decision making. The study failed to demonstrate a correlation between the grades of stenosis or the dural cross-sectional area and the initial ODI score or surgery outcomes. 

Soman et al. evaluated a qualitative grading system based on T2 weighted axial MRI images at the disc level to aid in selecting optimal treatment for multilevel lumbar spinal stenosis by a qualitative grading system using fluid rootlet ratio [20]. The study concluded that a grading system, in association with clinical evaluation, may be a useful tool in the decision-making process between conservative and surgical treatments of lumbar spinal stenosis. 

Weber et al. evaluated the correlation between the severity of stenosis and clinical presentation [21]. They found no clinical correlation between the radiological severity of lumbar spinal stenosis and the nature of the clinical findings, including disability, pain, and surgical outcomes.

Barz et al. identified the nerve root sedimentation sign as a distinct MRI finding to be associated with the diagnosis of lumbar spinal stenosis [11]. They found the nerve root sedimentation sign was always positive in patients with lumbar spinal stenosis and neurogenic claudication with a walking distance <200 meters and a dural sac <80 sq mm irrespective of any other clinical findings. A study by Macedo et al. evaluated the presence of a nerve root sedimentation sign in different groups of patients with degenerative lumbar spinal pathologies [22]. They found the incidence of a sedimentation sign was highest in patients with central stenosis (54%), followed by lateral recess stenosis (23%) and least in disc herniation (2%). In a patient subgroup with a dural cross-sectional area <80 sq mm and impaired walking capacity, the incidence of a sedimentation sign was higher (82%). Moses et al., based on the Spine Patient Outcome Research Trial (SPORT) cohort of patients with lumbar spinal stenosis without degenerative spondylolisthesis, identified a marginal, but higher surgical improvement in ODI scores in patients with a positive nerve root sedimentation sign [13]. Conversely, in another study by Barz et al., a positive nerve root sedimentation sign was not related to surgical outcomes [14]. The study by Fazal et al. indicated a sedimentation sign could be utilized in an objective assessment of MRI findings of lumbar spinal stenosis that may need surgical intervention [15].

Interspinous spacers are a more recent addition to the existent surgical management options in treating spinal stenosis. A systemic review by Kovacs et al. concluded different modes of surgical intervention, including interspinous spacers, were more beneficial than non-operative measures in the treatment of spinal stenosis [5]. A review by Wu et al. identified interspinous spacers to be an effective and less invasive option in the treatment of lumbar spinal stenosis [16]. However, the use of these devices was associated with higher re-operation rates and greater costs. A prospective, randomized controlled trial by Zucherman et al., comparing interspinous spacers versus standard conservative treatments, identified interspinous devices to be less invasive and an effective option for management of lumbar spinal stenosis [17]. In spite of numerous studies supporting the efficacy of this device, the lack of clarity for the indication of use, unfavorable results, and complications have limited the use of these devices. A review by Pintauro et al. highlighted the importance of identifying the correct indications for use to achieve optimal outcomes using interspinous spacer devices [23]. In addition, there was a concern for the long-term efficacy of these devices, especially from the earlier generations. A retrospective study by Gazzerri et al. evaluated the limitations and failures of interspinous spacers [24]. Their study identified factors that contribute to suboptimal outcomes using these devices.

The evolution of an objective methodology that prognosticates the outcomes of surgical intervention, especially with the use of interspinous devices in patients with lumbar spinal stenosis, would be greatly beneficial. It may lead to the development of additional objective guidelines for utilizing these devices in patients with clinical and radiological findings of lumbar spinal stenosis.

The study describes the nerve root sedimentation sign as a radiological indicator that possibly could assist with the surgical decision-making process as well as the prognostication of the outcomes in patients with symptomatic lumbar spinal stenosis. Although these findings may have a restricted applicability, the study discusses the scenarios where the use of nerve root sedimentation signs could be most relevant. In the future, it may be possible to utilize the nerve root sedimentation sign for predicting the outcomes of surgical techniques beyond interspinous devices in patients with lumbar spinal stenosis, but this would need validation with additional studies.

There were a few limitations in relation to this study. First, this study had a single intervention option with no comparative cohort. MRI image sequences from 40 patients were excluded due to incomplete images (N=24) or disagreement between the two reviewers (N=16). Although the study had a sufficient sample size (N=334), the subgroup analysis included only a fraction these patients; hence, these results could be statistically underpowered. Also, the study did not include additional measurements, such as canal diameters in the groups with and without a sedimentation sign. Hence, a comparative analysis with the conclusions from other studies cannot be provided. Consequently, this study does not strongly contradict or support the findings of other studies [22]. Another limiting factor is the lack of uniformity in MRI image quality. This was addressed by sub-classification of the images based on their quality. The classification of the images was devised by the senior authors and has not been validated; thus, adding a potential source of bias to the study. The assessment of the images was carried out by experienced fellowship-trained spine surgeons with a high inter-reviewer agreement, but this may make the study susceptible to an element of subjectivity and inter-observer bias. Although the lack of uniformity in the image quality was a concern, it also reflects the reality most clinicians face as a part of their clinical practice. Crucial treatment decisions may be made based on less than optimal images, due to a variety of factors beyond the clinician's influence. Lastly, in reference to the subgroup analysis that indicated a statistical difference in the improvement with postoperative ODI scores between the two groups, it should be noted that these differences may not have a clinical relevance.

## Conclusions

This study demonstrated satisfactory post-intervention improvement in lumbar spinal stenosis patients with and without nerve root sedimentation signs treated with interspinous spacers. Subgroup analyses indicated that the MRI image quality and nerve root sedimentation sign certainty of determination appear to be factors that may aid with planning the surgical management of lumbar spinal stenosis. The highest grade of the MRI image quality and certainty of nerve root sedimentation sign may be an indicator, albeit weak, for better clinical improvement, and similarly the positive nerve root sedimentation sign group correlating to greater ODI improvements. The lack of a validated classification system for the determination of nerve root sedimentation along with the element of inter-observer bias in the assessment are definitive limitations. In essence, the nerve root sedimentation sign, along with other findings, may have a certain value in predicting the success of surgical treatment with the use of interspinous devices in patients with lumbar spinal stenosis.
